# Gynaecological morbidities among married women and husband’s behaviour: Evidence from a community‐based study

**DOI:** 10.1002/nop2.660

**Published:** 2020-10-17

**Authors:** Tazeen Saeed Ali, Neelofar Sami, Adil Ali Saeed, Parveen Ali

**Affiliations:** ^1^ School of Nursing and Midwifery Aga Khan University Karachi Pakistan; ^2^ Department of Community Health Sciences Aga Khan University Karachi Pakistan; ^3^ Intern of School of Nursing and Midwifery Aga Khan University Karachi Pakistan; ^4^ University of Sheffield Sheffield UK

**Keywords:** gynaecological morbidities, intimate partner violence, psycho‐social impact

## Abstract

**Aim:**

To determine the association between gynaecological morbidities and IPV among married women specifically, with attention to the attitudes of the husband and the degree of satisfaction in a marital relationship.

**Design:**

Cross‐sectional study design.

**Methods:**

Data were collected using face‐to‐face interviews with married women aged 15–49 years, living in selected communities. Information was collected on demographic characteristics, gynaecological morbidities and IPV using a self‐developed tool. Descriptive and inferential statistics were used to analyse the data.

**Results:**

Logistic Regression showed a significant association between physical violence and burning micturition, increased urinary frequency, constant dribbling of urine, genital ulcers, lower abdominal pain, vaginal discharge and painful coitus (OR: 1.41–1.84). A significant association between sexual and psychological abuse was also found with burning micturition (OR: 1.41) and dribbling of urine (OR: 0.12). Since gynaecological morbidities can have a serious effect on the psychological, physical well‐being, and the social status of women in Pakistan; effective interventions are imperative in dealing with their symptoms and decreasing their emergence.

## 1 BACKGROUND

Sexual health is an integral part of an individual's life. Sexual fulfilment is not only a physiological need like sleep, hunger, and thirst but is also considered a spiritual need. Sexual health not only affects the quality of life of an individual but of their intimate partner. Sexual intimacy is an important part of a marital relationship and an inability of one partner in meeting the needs of another can jeopardize marital relationships and bonding between intimate partners. There is a positive correlation between sexual satisfaction and marital commitment. Sexual health issues affecting men and women are equally problematic; however, there are many different issues that can affect women's sexual and reproductive health, and these could be due to menstrual problems or a consequence of pregnancy and childbirth (Dheresa et al., 2017).

Issues regarding sexual health are a major contributor to health problems globally, particularly in low‐income countries such as Pakistan. Poor reproductive health among women and other resulting conditions contribute significantly to the burden of disease, yet meagre data are available to know exact impact of the problem. Pakistan is not an exception to this since studies have evidenced a high prevalence of conditions affecting the sexual health of women. A study from a squatter settlement in Karachi showed a high perceived prevalence of morbidities including menstrual disorders (45.3%), pelvic inflammatory disease (12.8%) and urinary tract infection (5.4%) among women (Sajan & Fikree, 1999). The poor health of women due to various conditions and associated complications usually compromises their ability to perform routine daily responsibilities efficiently. As mentioned above, it also can have an impact on the sexual relationship with their spouse and (Dheresa et al., 2017), consequently, can have an impact on their marital relationship. In a country like Pakistan, sexual health is a taboo topic, people may not talk about it or seek help at the right time and gradually the issue can start having an impact on life as a whole. Husbands may develop a non‐supportive attitude contributing to increased likelihood of marital conflict and even intimate partner violence (IPV) (Gracia, 2014).

Worldwide, IPV has been identified as a common problem with high prevalence varying from 08% to 61% approximately (Malik, Shabila, & Al‐Hadithi, 2016). In Pakistan, the prevalence of physical, sexual and psychological violence among women is reported as 57.6%, 54.5% and 83.6%, respectively with the husband as the perpetrator in most cases (Ali et al., 2011). Studies have shown that IPV has been associated with adverse health conditions including gynaecologic problems. These include poor general health, injury, chronic pain, acquired immune deficiency syndrome (AIDS), sexually transmitted infections (STI), vaginal bleeding and lacerations, unwanted pregnancy, dyspareunia, urinary tract infections (UTI) (Abramsky et al., 2011; Watts & Mayhew, 2004) and reproductive tract infections (RTI). Additionally, IPV results in mental health problems such as anxiety and depression (Ali et al., 2011), substance abuse and suicidal ideation.

While we understand that gynaecological conditions could affect the sexual life of men and women and, therefore, have an impact of marital satisfaction, not much research is conducted to explore the association between gynaecological morbidities and marital conflicts and/or IPV. A comprehensive literature search could only identify two studies that explored the impact of gynaecological cancer on patients and their partners (Iżycki, Woźniak, & Iżycka, 2016; Ratner et al., 2010). The present study, therefore, was conducted to fill the research gap by exploring the impact of gynaecological problems in women and the impact of their husband's attitude towards them and their marital life. The study also aimed to determine an association between gynaecological symptoms and different forms of violence (IPV) among married women, in urban Karachi.

## 2 METHODOLOGY

For this cross‐sectional study, we recruited married, non‐pregnant women aged 15–49 years registered with the Lady Health Workers (LHWs) programme in three selected union councils of Karachi, Pakistan. The LHW programme is a national initiative implemented by the Pakistani government and provides a variety of doorstep family planning services and access to resources that can improve women's reproductive health (Mumtaz et al., 2013). The programme aims to allow women access to healthcare systems where they may otherwise have been secluded in a largely patriarchal society. Data for this study were collected by the LHWs of the National Program for Family Planning (FP) and Primary Health Care (PHC). The National Program for FP and PHC aims to provide health services to underdeveloped, rural and peri‐urban communities at their doorstep. The LHWs provide health services through monthly home visits. Each LHW is responsible for providing services to 150 households. Considering Karachi, it is the metropolitan city in the province of Sindh with a population of approximately 22 million (Ali, et al., 2011). It occupies an area of 3,530 square kilometres. Karachi is divided into six districts and 18 towns, sub‐divided into 178 union councils (UCs). These UCs are the core element of the local government system. Detailed information about city demographics is published elsewhere (Sami et al., 2015).

The women selected for the study were required to report any form of gynaecological morbidity. They were asked about their husband's attitude towards them and their perceived marital satisfaction. Women who reported facing IPV were asked about any support required and were referred to institutions providing free services and resources for victims.

The study was approved by the Aga Khan University Ethical Review Committee. Before recruitment, each participating woman was provided with the appropriate information about the study and the process of enrolment. The female participants also needed to meet the requirements of the study which included a general physical and per vaginal examination and sample collection to investigate RTIs and STIs. The participants were aware of the incentive (i.e., if found positive for any disease during the course of screening, they shall receive treatment free of cost). A written informed consent was also obtained from each woman.

### 2.1 Sample

A multi‐stage sampling technique was used. Initially, purposive sampling was used to select 3/78 UCs out of in Karachi that included the LHW programme. This helped minimize the risk of sampling bias as women were recruited from all UCs. In the second stage, a random sampling technique was used to identify and recruit study participants. This involved developing a list of all households followed by use of computer‐generated numbers to identify and select currently married, non‐pregnant women aged 15–49 years. Households (*N* = 350) in each UC were selected and LHW confirmed the age of the woman who was married and not pregnant (15–49 years) by visiting each household. Where there was more than one married, non‐pregnant woman in a household, one woman was selected randomly (their names were written on a piece of paper and one was randomly selected). If the woman approached for the study did not consent or was not available, the next household was considered. If the selected woman was <18 years of age, consent was taken from the husband or any other older family member.

The sample size was calculated on the basis of the expected prevalence of selected STIs in comparable surveys conducted in Pakistan, Bangladesh, India, Lebanon and other neighbouring countries. We required 945 participants and assuming 10% refusals, the total sample size was calculated to be 1,039. It was rounded to 1,050, with 350 women from each UC (Bhatnagar & Khandikar, 2013; Deeb et al., 1997; Tulasi & Babu, 2018). A total of 1,050 women were invited to participate and 1,002 women consented, resulting in a response rate of 95.4%. The main reasons for refusing to participate in the study included an inability to get permission from the family or husband and the fear of being diagnosed with a disease. Of the 1,002 women interviewed, a total of 945 underwent physical and pelvic examinations which provided samples for laboratory investigations.

### 2.2 Operational definitions of variables

The World Health Organization (WHO) (2013) defines IPV as any behaviour within an intimate relationship which causes physical, psychological or sexual harm to intimate partners. IPV can occur in a heterosexual and in a same‐sex relationship. It can also be perpetrated by women against male or female partners. However, women remain the most common victim of IPV. A husband's negative outlook is the husbands actions or reaction towards his wife due to gynaecological morbidities that made women uncomfortable, especially in engaging in sexual intimacy (Mahapatro et al., 2012).

### 2.3 Data collection

Trained interviewers collected information from each participant using a questionnaire that explored the participant's menstrual and obstetric history, perceived gynaecological symptoms, and knowledge and experience of IPV. Each woman then underwent a general physical examination, as well as a pelvic examination, and had her samples collected for laboratory investigations.

### 2.4 Analysis

The data were double‐entered using Epi‐Info. The statistical analyses were conducted using the Statistical Package for Social Sciences (SPSS) version 19. The prevalence of infection (with 95% confidence intervals) was determined using confirmatory laboratory test results. In addition to descriptive analysis, logistic regression was used to assess the association of gynaecological morbidities and the attitude of husbands towards their wives.

To analyse the demographic data, women were categorized into seven groups based on their diasporic ethnicity: Urdu; Sindhi; Balochi; Punjabi; Pashto; Hindko; and Siraiki. To assess their literacy status, the following parameters were used: illiterate, primary education (Grade 1–5), secondary education (Grade 6–10), and higher education (11 and above, including postgraduation). The socio‐economic status of the participants was also analysed using various proxies such as monthly income, source of water supply, ownership of the house and other items used for daily living: such as radio, TV, refrigerator etc. The socio‐economic status for each participant was estimated by giving a score for each item and adding the total scores. We also conduced multivariate analysis for educational status of the husband, duration of the marriage, and number of children with each morbidity with husband's attitude and found all insignificant results at 5% alpha level.

## 3 RESULTS

### 3.1 Demographics

Most participants were Urdu and Punjabi speaking (20% each), followed by Hindko (14.6%), Pashto (13.5%), Balochi (13.3%), Sindhi (13.2%), Siraiki (4.3%), and others (0.5%). Nearly 44% of the women were illiterate. While one‐fifth had completed 5 years of schooling, nearly one‐third had completed 10 years. A few had 14 years of education (5.2%) and a postgraduate degree (1.0%). We also asked women about the educational status of their husbands revealing 26.7% of husbands as illiterate; 15.3% had completed primary education; 42.4% had received education up to class 6–10 and 14.0% had received education up to class 11–14. Looking at their socio‐economic statuses, most (79.7%) of our respondents lived in well‐constructed houses and reported using tap water available inside the house. Of the respondents 53.4% owned their house, 45.9% lived in a rented house while the remaining few (0.7%) had other living arrangements as shown in the table 1. Most owned a fan (97.7%) and an iron (95.9%). A considerable portion of the population owned a TV (75.6%), a washing machine (71.0%), and a refrigerator (53.7%). Some also had a radio (23.3%), a motorcycle (16.3%), bicycle (11.1%) a video player (6.9%) and a car (3.2%). We assessed the socio‐economic status of our respondents using the data collected in Table 1 and analysed it using the point scoring system. Using this practice, we divided our population into three categories. More than half (51.5%) of the study sample belonged to a lower socio‐economic status, 25.8% belonged to a middle socio‐economic status and 22.8% belonged to a higher socio‐economic status. Of 1,002 women, 15.7% (*N* = 157) reported experiencing physical abuse, 19.7% (*N* = 197) reported sexual abuse and 43.4% (*N* = 435) reported psychological abuse during the past year. Of the 1,002 participants, 13.8% had no pregnancy, 46% had one to four children, 27.5% had five to eight children, and 12.7% had more than eight children (Table 1).

**Table 1:** Socio‐demographic characteristics of participants

Characteristics	*N*	%
Language spoken at home		
Urdu	210	21.0
Punjabi	344	134.4
Pashto	135	13.5
Balochi	133	13.3
Sindhi	175	17.5
Education level of respondent		
Illiterate/Can read only the Holy Book	439	43.8
Class 1–5 ( Primary)	206	20.6
Class 6–10 ( secondary)	295	29.4
Higher education (Class 11−14/ Postgraduation)	62	6.2
Respondents’ work status		
Yes	108	10.8
No	894	89.2
Respondent's occupation		
White collar jobs	37	37.0
Blue collar job	63	63.0
Husband's education		
Illiterate	268	26.7
Class 1–5	153	15.3
Class 6–10	425	42.4
Education higher than 10th grade	171	17.1
Husband's occupation		
White collar job	160	16.0
Blue collar job	832	83.2
Construction of the house		
Well‐constructed	786	78.4
Not Well‐constructed	141	14.1
Kacha‐Pakka	75	7.5
Source of water supply to the house		
Tap water from inside the house	799	79.7
Tap water from outside the house	203	20.3
Latrine facility		
Flush system W.C	770	77.0
Without flush system W.C	230	23.0
Ownership of the house the respondent lived in		
Yes	535	53.4
No	467	46.6
Socio‐economic status		
High SES	228	22.8
Middle SES	259	25.8
Lower SES	515	51.4
Reported violence's		
Physically abused	157	15.7
Sexually abused	197	19.7
Physiological abused	435	43.4

### 3.2 Morbidities

In this study, a high proportion of women reported vaginal discharge (65.4%), lower back pain (56.2%), lower abdominal pain (51.4%) and dyspareunia (44.2%) (Table 2). Around two fifths of the women reported an increased frequency of general micturition and burning micturition, as well as irritation over the genital area. Urinary incontinence was reported by 30% of the women. Other, less frequently reported morbidities included genital vesicles (6.7%), constant dribbling of urine (6.4%), postcoital bleeding (4.9%) and genital ulcers (4.1%).

**Table 2:** Showing Gynaecological morbidities with Husbands attitude and marital life experience, Karachi Pakistan

Morbidities	Number of women reported morbidity *n* (%)	Husbands attitudinal problem due to morbidity	Marital life effected due to morbidity
Virginal discharge	656 (65.4)	4 (0.6)	107 (16.3)
Backache	563 (56.2)	3 (0.5)	76 (13.4)
Lower abdomen pain	515 (51.4)	4 (0.7)	75 (14.5)
Painful coitus	446 (44.2)	407 (91.2)	305 (68.3)
Increased frequency micturition	390 (38.9)	353 (90.5)	35 (8.9)
Burning Micturition	340 (33.4)	308 (90.5)	43 (12.6)
Irritation at genital area	286 (28.5)	2 (0.6)	54 (18.8)
Dribbling of urine with coughing sneezing and laughing	266 (26.5)	237 (89.0)	20 (7.5)
Genital vesicles	67 (6.7)	1 (1.4)	15 (22.3)
Constant dribbling of urine	64 (6.4)	51 (79.6)	8 (12.5)
Genital ulcers	41 (4.1)	00 (0%)	7 (17.0)
Prolapse	240 (23.9)	51 (21.2)	20 (8.3)

With regard to the gynaecological morbidities painful coitus (68.3%), genital vesicles (22.3%), irritation at genital area (18.8%), genital ulcers (17%), and vaginal discharge (16.3%) were reported to have negatively affected their sexual and therefore marital relations. Though, to a lesser extent, other issues such as lower abdomen pain (14.5%), backache (13.4%), burning micturition (12.6%) and constant dribbling of urine (7.5%) were also reported to have affected the marital relationship negatively.

Women believed that their husbands had a negative outlook concerning painful coitus (91.2%) and urinary morbidities such as increased urinary frequency, burning micturition and dribbling urine with coughing, sneezing, and laughing. Women considered their husbands were less concerned about the women's complaints of lower abdominal pain, vaginal discharge, irritation at genital area, backache and genital ulcers.

**FIGURE 1:**
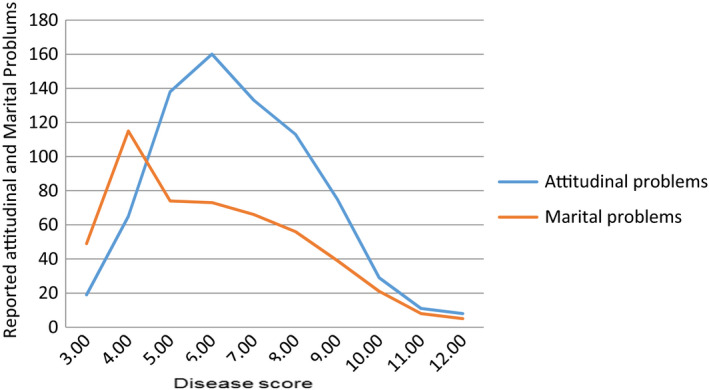
Showing the relationship between husband's attitude and affected perceived marital relationship due to gynacological morbidites. *n* = 1,002

The number of symptoms of gynaecological morbidities a woman reported was directly proportional to the negative outlook, husbands had about their wives’ conditions. As a result, the negative viewpoints contributed to a negative impact on the marital relationship. As shown in Figure 1, of the 1,002 participants, women who reported three morbidities, received less negative views from their husbands and had a better perceived marital relationship (i.e., less marital problems). However, as morbidities increased to four and above, the husband's negative attitude increased till it reached to the level of the six reported morbidities. As after seven and above, the attitude of the husband became rather supportive. Here, marital problems were reported as being far more than attitudinal issues. However, as the number of symptoms of morbidities increased from seven onwards, the husband's negative attitude towards their wives and marital problems decreased. We see a bell‐curve trend which correlates to the cultural attitudes in Pakistan, where when a person becomes severely sick, they have a multitude of support from family, in‐laws and especially the husband, who becomes her major caretaker.

We also found that the higher the husband's educational status (*X* ^2^ *p* value: <.001), the longer the duration of the marriage (*X* ^2^ *p* value: <.001) and the greater the amount of children a couple had(*X* ^2^ *p* value: <.001), attitudes towards women with morbidities were less negative and a better marital life was perceived. When the morbidity score was <3, problems with negative attitudes and marital problems were very low (almost zero).

This study also looked at the association of symptoms of gynaecological morbidities, reported by the women, with physical, sexual, and psychological violence (Table 3). We found that violence was related to a negative outlook on the husband's part towards his wife, and an overall less satisfactory martial life. Specifically, the less supportive the husband was, the higher the incidence of violence, especially physical and sexual violence. The symptom of “constant dribbling of urine” was significantly associated with physical, and psychological violence ([OR: 2.88; 95% CI: 1.65–5.00], [OR: 2.13; CI: 1.27, 3.58], respectively). An increased frequency of general micturition was also associated with physical and sexual violence ([OR: 1.58; CI: 1.13–2.23], [OR: 1.41; CI: 1.03, 1.93] respectively). There was a statistically significant association of dyspareunia with physical (OR: 1.84; CI: 1.30–2.60) and psychological violence (OR: 1.57; CI: 1.22–1.01). No significant association was found between dyspareunia and sexual violence (OR: 0.77; CI: 0.56–1.66). Sexual and physical violence was also found to be associated with genital ulcers ([OR: 2.96; CI: 1.51, 5.79]), lower abdomen pain (OR: 1.41; CI: 1.01, 1.99) and vaginal discharge (OR: 1.53; CI: 1.05, 2.25). Dysmenorrhoea was also associated with sexual violence (OR: 1.69; 1.13, 2.51).

**Table 3:** Univariate analysis of association of gynaecological symptoms with different forms of violence among married women, Urban, Karachi *n* = 1,002

Reported morbidities	Reported physical violence	Reported sexual violence	Reported psychological violence
Urinary morbidity			
Burning micturition	1.42 (1.03, 2.01)	0.82 (0.58, 1.14)	1.06 (0.817, 1.38)
Increased frequency of maturation	1.58 (1.13, 2.23)	1.41 (1.03, 1.93)	1.14 (0.88, 1.47)
Constant dribbling of urine	2.88 (1.65, 5.00)	0.12 (0.03, 0.50)	2.13 (1.27, 3.58)
Dribbling of urine with coughing sneezing and laughing	1.22 (0.84, 1.77)	0.99 (0.69, 1.41)	0.99 (0.74, 1.31)
Coital morbidity			
Dyspareunia	1.84 (1.30, 2.60)	0.77 (0.56, 1.06)	1.57 (1.22, 1.01)
Postcoital bleeding	1.80 (0.92, 3.54)	0.66 (0.29, 1.51)	0.89 (0.49, 1.60)
Sexually transmitted infections			
Irritation on genital area	1.16 (0.80, 1.68)	1.12 (0.79, 1.57)	1.07 (0.81, 1.42)
Genital ulcers	2.96 (1.51, 5.79)	0.31 (0.09, 1.02)	2.10 (1.10, 3.98)
Genital vesicles	1.46 (0.79, 2.70)	0.98 (0.52, 1.83)	1.06 (0.64, 1.74)
Backache	0.85 (0.60, 1.20)	0.98 (0.71, 1.34)	0.97 (0.75, 1.25)
Lower abdomen pain	1.41 (1.00, 1.99)	0.94 (0.69, 1.29)	1.18 (0.92, 1.52)
Vaginal discharge	1.53 (1.05, 2.25)	0.94 (0.68, 1.31)	1.22 (0.94, 1.59)
Prolapse			
UV prolapse	1.61 (1.10, 2.33)	0.68 (0.46, 1.01)	1.21 (0.90, 1.62)
Menstrual morbidity			
Irregular menstrual cycle during last 3 months	1.30 (0.91, 1.84)	1.25 (0.91, 1.73)	1.20 (0.92, 1.56)
Scanty periods during last 3 months	0.95 (0.60, 1.50)	1.10 (0.73. 1.65)	1.30 (0.94, 1.81)
Heavy Periods during last 3 months	0.89 (0.38, 2.06)	0.93 (0.61, 1.42)	1.21 (0.87, 1.69)
Dysmenorrhea	1.49 (0.97, 2.28)	1.69 (1.13, 2.51)	0.97 (0.73, 1.29)

## 4 DISCUSSION

The burden of gynaecological morbidities is significant. The highest reported morbidities were vaginal discharge, backache and lower abdominal pain. All these symptoms signify the presence of an infection in the genital tract among females. However, these problems have shown little to no effect on the husband's attitude towards his wife and their marital relationship. There is a possibility that these morbidities only caused problems to the women and, therefore, the husband was not affected. For example, a study on complaints about vaginal discharge explained that the husband may not take notice of his wife's morbidity causing little effect on his attitude towards her (Patel et al., 2005).

The morbidities resulting in a husband's pessimism and subsequent marital life issues were those linked with sexual needs of the husband such as painful coitus, increased frequency of micturition, burning micturition and stress incontinence. This could be explained by the idea that if a woman has urinary incontinence, dribbling problems or foul‐smelling vaginal discharge (Stephenson, Elfstrom, & Winter, 2013), for example, it could contribute to an unpleasant feeling during intercourse for husband and wife leading to an unsatisfactory sexual encounter. The unfulfilled sexual needs, then contribute towards the negative attitudes and possibly IPV of their husbands. Painful coitus can also have a negative impact on the sexual satisfaction of a husband and wife, contributing to further stress on the marital relationship (Ali, et al., 2011). A study conducted in India revealed that the improved educational status of the husband (Singh et al., 1998) was associated with less negative attitudes from the husband and a perceived better martial life. The longer duration of the marriage and having more children were also a significant factors associated with husband's attitude supportive attitude and a wife's perception of a better marital life (Thankain et al., 2015). However, in our multivariate model, it remains non‐significantly associated with these variables suggesting SES does not make any difference. It is the husband's satisfaction level for sexual contact which affects his attitude with his wife.

Women's gynaecological morbidities and husbands’ negative attitude along with a lack of empathy could lead to the couple sharing an unhealthy marital life. The woman is then unable to perform her duties and unable to concentrate on her work (Ali, 2011). Therefore, her family and husband may disregard her. Existing literature suggests that, due to the lack of support from her husband, the woman often shows symptoms of anxiety, depression, migraine and psychological problems (Pardeshi et al., 2017; Sami et al., 2015). These have potential to become severe and lead to instances of suicide (Ali, et al., 2011). A population‐based study among married women showed that in the total population of women, mental health issues were prevalent. Women subjected to any form of violence reported poorer mental health than unexposed women (Whitaker, Orzol, & Kahn, 2006).

The strongest associations were found between constant dribbling of urine, genital ulcers, UV prolapse and physical violence; dysmenorrhea and sexual violence; and constant dribbling of urine 2.13 (1.27, 3.58), genital ulcers and psychological abuse. Due to these morbidities, women are exposed to all forms of violence that often lead to depression in addition to pain. Women in a patriarchal society like Pakistan face an increased risk for both pain and depression due to many causes in addition to exposure to violence, yet little is known about the frequency and implications of comorbid pain and depression among women (Zahidie & Jamali, 2013).

Similar studies also indicate that morbidities like dyspareunia, urinary incontinence and UTIs are prevalent in society and are not considered important to treat in women. In some cases due to the foul smell, it is less acceptable by the husbands and close family members. Dyspareunia, a form of sexual dysfunction, can significantly affect quality of life and cause relationship difficulties among couple. (Judd et al., 2012; Revicky et al., 2012).

Furthermore, UV prolapse, a significant finding in our study, can lead to painful coitus which further intensifies the husband's negative attitudes towards sex and the marital relationship. This causes her to be exposed to physical, sexual, and psychological violence. Multiple reasons can be considered. First, if a pregnant woman is delivering at home, she can experience complications, and with the lack of proper care, she can experience prolapse later. Second, she can experience poor nutrition. Third, in the present set‐up, women also look after the children; both physically and educationally, in addition to facing any family issues. Besides this, they also have to perform heavy lifting jobs like ploughing fields and bringing water from far fledged areas. In rural areas, where these tasks are prevalent, along with a limited access to healthcare, gynaecological morbidities can worsen (Walker & Gunasekera, 2011).

In this study, we have identified that, as the morbidities increase to score 7 and above, the husband's overall attitude towards his wife and, consequently, their perceived marital life improves. In Pakistan, most citizens are Muslim and are culturally sensitive when it comes to those who are sick. Sick people are supported by the society and it is expected that husbands take care of their sick spouse. The issue here is that society seems to accept a woman's illness when it becomes severe and she is bed‐ridden as opposed to when she is able to take care of the home and chores with bearable symptoms. Our survey indicates that the overall family attitude is supportive when a woman is diagnosed with a gynaecological morbidity. Although this phenomenon is not published, comparison with the gynaecological morbidities, the literature does support that at certain times a husband's attitude is more supportive when his wife is very sick.

In Pakistani society, the woman is not only responsible for multi‐tasking with various relationships and household chores, but also tends to face the brunt of all issues, whether she is responsible or not. Her health soon becomes her last priority, both physically and emotionally. She is a constant target of humiliation and domestic violence. However, much of her situation is a result of her lack of awareness about issues involving women's health and her inability to take a stand for her own rights, therefore education is key to her empowerment (Malik & Courtney, 2011; Noureen, 2015).

## 5 LIMITATIONS

Certain limitations in the study may have affected the results. This includes sensitive questions posed to the participants about gynaecological symptoms, spousal behaviour, and acts of domestic violence which may not be culturally acceptable for women to answer truthfully. Efforts were made to interview women privately. However, the level of comfort with the arrangements of the study made remained a concern for a women to express completely, as this was a very personal topic. The study was also conducted in urban squatter settlements of Karachi and results may not be applicable to all areas such as rural areas. Also, perceptions and experiences about gynaecological morbidities were only taken from the woman's perspective. It is equally important to learn about the husband's perspective, which was not addressed in this study. Another limitation, in sampling, was that some participants were under 18 years of age. However, the sample had only six participants who were under 18 which accounted for about 0.6% of the study. This did not skew the results significantly because most of the women were 18 years or older. Also, the study has not developed a multivariate model that would address confounding variables like parity history; however, in the future, this would be considered to adjust for confounding variable. Yet these data provide important findings which have never been shared in South Asian countries.

## 6 RESEARCH IMPLICATIONS

This study indicates the need to identify gynaecological morbidities in women of reproductive age at the primary care level so that their burdens can be addressed in a timely manner, complications are prevented and social implications can be lessened. This does not only have relevance to a women's current health, but also for their future risks to develop other complications such as HIV. Healthcare providers can then learn and allow for the promotion of proper training to better treat these morbidities physically, emotionally and socially. Overall, it will create a culture of awareness among communities and healthcare providers allowing women to be better understood when looking at how morbidities have an impact on their lives.

## 7 CONCLUSION

Women living in a largely patriarchal culture are not only experiencing several gynaecological morbidities, but are also facing their husbands’ negative attitude towards them, which is affecting their marital life. Violence against women, namely IPV, is shown to correlate with women having gynaecological morbidities and poor reproductive health. Specifically, the marital duties that they may not be able to perform due to discomfort can be the cause of an unsatisfactory marital life and put a woman's well‐being at risk. Hence, if a woman becomes mildly sick, she does not get support from her husband. We hope this study can be used to help form the definition around gynaecological morbidities and rely some implications they have on a woman's daily life. Therefore, taking this study into consideration, we recommend mass awareness programmes that integrate education for husbands and the entire family, along with counselling when women who experienced abuse are identified in clinical or hospital settings. Also, public education programmes supporting women's health and their rights should be encouraged, in educational institutions and among the general public, with an emphasis on women's gynaecological and reproductive health. Furthermore, government policies should be revised and laws should be implemented to safeguard women's health and safety.

## CONFLICT OF INTEREST

None of the author is having any conflict of interest.

## AUTHORS CONTRIBUTIONS

TSA: Main idea, Co‐investigator of the study, field supervision, data analysis and first draft write up. NS: Main idea, principal investigator of the study and field supervision and assisted in first draft write up. AAS: Contributed in data analysis, literature search, and rewriting of the manuscript. PA: Reviewed the drafts before submission.

## Data Availability

The data will be provided on request.
